# The Arabidopsis *altered in stress response2* is Impaired in Resistance to Root and Leaf Necrotrophic Fungal Pathogens

**DOI:** 10.3390/plants8030060

**Published:** 2019-03-11

**Authors:** Louise F. Thatcher, Karam B. Singh

**Affiliations:** 1 CSIRO Agriculture and Food, Centre for Environment and Life Sciences, Wembley, Western Australia 6913, Australia; Karam.Singh@csiro.au; 2 The Institute of Agriculture, The University of Western Australia, 35 Stirling Highway, Crawley, Western Australia 6009, Australia; 3 Centre for Crop and Disease Management, Department of Environment and Agriculture, Curtin University, Bentley, Western Australia 6102, Australia

**Keywords:** disease susceptibility, GSTF8, necrotroph, jasmonate, At2g31320, At2g31400, At2g31990, At2g33170, At2g35170, At2g35740, At2g36420, At2g36835, At5g53060, CPL1, At4g21670

## Abstract

The *Arabidopsis thaliana* *Glutathione S-transferase Phi8* (*GSTF8*) gene is recognised as a marker for early defence and stress responses. To identify regulators of these responses, a forward genetic screen for Arabidopsis mutants with up-regulated *GSTF8* promoter activity was conducted by screening a mutagenized population containing a *GSTF8* promoter fragment fused to the luciferase reporter gene (*GSTF8:LUC*). We previously identified several *enhanced stress response* (*esr*) mutants from this screen that conferred constitutive *GSTF8:LUC* activity and increased resistance to several pathogens and/or insects pests. Here we identified a further mutant constitutively expressing *GSTF8:LUC* and termed *altered in stress response2* (*asr2*). Unlike the *esr* mutants, *asr2* was more susceptible to disease symptom development induced by two necrotrophic fungal pathogens; the root pathogen *Fusarium oxysporum,* and the leaf pathogen *Alternaria brassicicola*. The *asr2* allele was mapped to a 2.1 Mbp region of chromosome 2 and narrowed to four candidate loci.

## 1. Introduction

Crop damage caused by pathogen and pest attack costs the global economy billions of dollars annually, with the Food and Agriculture Organization of the United Nations (FAO) estimating global annual yield reductions in the range of 20% to 40% [1]. The identification of disease resistance-associated genes with quantitative effects and an understanding of their function is a necessity to mitigate the ongoing effects of biotic stresses.

A large gene family associated with responses to an array of biotic stresses are the *Glutathione S-transferases* (GST) [2,3]. Through a combination of catalytic or non-catalytic functions, repertoires of GST protein products function in response to pathogen attacks by regulating oxidative bursts or the accumulation of defence-related metabolites [4]. In addition to involvement in biotic stress, some plant GSTs are also well known for their roles in response to abiotic stresses and the detoxification of xenobiotic compounds such as herbicides [5]. Of the 55 members in Arabidopsis, the *GSTPHI8* (*GSTF8*) gene is comparatively well studied and has emerged as a marker gene for early stress and defence responses [3,6,7,8]. Its expression is induced by multiple biotic elicitors including of fungal or bacterial origin, and phytohormones or signalling molecules such as salicylic acid (SA) or H_2_O_2_ [6,7,9,10,11,12,13].

Based on the well-characterised *GSTF8* expression profile [3,10,13,14], an Arabidopsis line containing the *GSFT8* promoter linked to the *Firefly Luciferase* reporter gene (*GSTF8:LUC*) has been used to non-invasively monitor a plant’s stress status and response to defence cues such as to plant hormones or challenge with fungal pathogens [6,11,12,15]. Coupling this line with a mutagenesis approach facilitated the discovery of genes involved in the positive or negative regulation of stress responses and the discovery of mutants altered in resistance to pathogens or pests [7,8,16]. One mutant termed *disrupted in stress responses1* (*dsr1*) encodes a positive regulator [7]. It exhibited a loss of salicylic acid (SA)-inducible *GSTF8:LUC* activity and increased susceptibility to several fungal and bacterial pathogens. The causal mutation results in a single amino acid change in a subunit of the mitochondrial energy machinery (complex II subunit SDH1-1), causing a reduction in induced reactive oxygen species (ROS) production from mitochondria.

Complementing the discovery of *dsr1*, several mutants with constitutive *GSTF8:LUC* expression were also discovered and designated as an *enhanced stress response* (*esr*). Three mutants (*esr1-1*, *esr1-3*, *esr1-4*) encoded alleles of the K homology (KH) domain containing RNA-binding protein At5g53060 and conferred increased resistance to the root-infecting fungal pathogen *Fusarium oxysporum* [8]. *Fusarium oxysporum* is considered a hemibiotrophic pathogen, living part of its life as a biotroph (a pathogen that derives nutrients from living host cells), and the other part, often associated with the later stages of infection, as a necrotroph (a pathogen that derives nutrients from dead cells) [17]. The *esr1* mutations conferred a STOP codon substitution or splicing defects at splice site junctions. Global transcriptome analysis of the *esr1-1* mutant compared to wild-type plants identified altered expression of genes involved in responses to biotic and abiotic stress [8]. A second negative defence-regulator, whose protein product interacts with ESR1/KH-domain, was identified from the same screen. Two mutant alleles of this gene, *Enhanced Stress Response3/RNA Polymerase II C-Terminal Domain (CTD) Phosphatase-Like1* (*CPL1*) (At4g21670) were cloned and also result from an induced stop codon change or splicing defect [16]. The *cpl1-7* (*esr3-1*) and *cpl1-8* (*esr3-2*) mutants displayed strong resistance to *F*. *oxysporum,* as well as increased resistance to the leaf necrotrophic fungal pathogen *Alternaria brassicicola* and to an aphid pest. Global transcriptome analysis of *cpl1-8* compared to wild-type plants identified up-regulated expression of genes related to defence and oxidative stress/redox state processes [16].

Here we extend on our analysis of mutants from the *GSTF8:LUC* mutagenesis screen and isolate a second class of constitutively expressing *GSTF8:LUC* mutant. This mutant termed *altered in stress response2* (*asr2*) differs from the constitutive *GSTF8:LUC esr1* and *cpl1* mutants and confers increased susceptibility to both the root fungal pathogen *F. oxysporum* and the leaf fungal pathogen *A. brassicicola*, suggesting *ASR2* encodes a positive regulator of defence responses. The causal mutation was narrowed to four candidate loci not previously implicated in resistance to fungal pathogens.

## 2. Results

### 2.1. Identification of asr2

**Figure 1:**
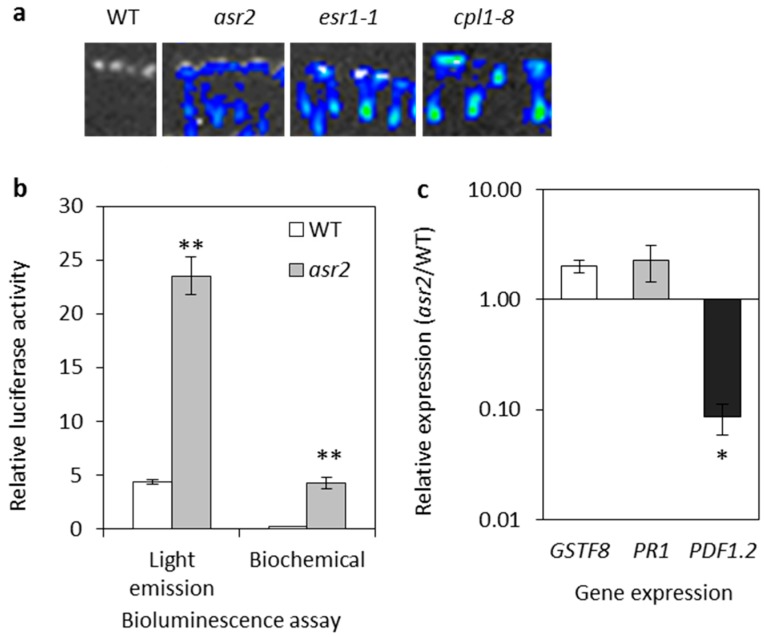
*asr2* causes basal hyper-expression of *GSTF8::LUC* and altered defence marker gene expression. (**a**) Comparison of basal *GSTF8:LUC* expression in four-day-old wild-type (WT), *asr2*, *esr1-1,* and *cpl1-8* (*esr3-2*) seedlings. (**b**) Quantification of bioluminescence via in vivo light emission (relative light units/seedling; values are averages ± SE (*n* = 30) from four-day-old seedlings) and in vitro biochemical assays (units/20sec/mg protein; values are averages ± SE (*n* = 30) from nine-day-old seedlings). (**c**) *GSTF8*, *PR1,* and *PDF1.2* expression in seven-day-old seedlings (values are averages ± SE of two biological replicates consisting of pools of 20 seedlings). Gene expression levels are expressed logarithmically relative to the normalised expression levels in WT plants. For normalisation, the internal control *EF-1* gene was used. Asterisks indicate values that are significantly different (** *p* < 0.01, * *p* < 0.05 Student’s *t*-test) from WT.

From our previous screen that identified the *esr1* and *cpl1* (*esr3*) mutants [8,16], a further 40 constitutively expressing *GSTF8:LUC* mutants were identified including one M2 mutant labelled *asr2* that, following *esr1* and *cpl1,* had the next highest levels of basal promoter expression (Figure 1a). Reciprocal crosses between *asr2* and *esr1-1*, *cpl1-7* (*esr3-1*) or *cpl1-8* (*esr3-2*), and assessment of complementing constitutive luciferase phenotypes in F_1_ populations determined *asr2* was not an allele of either e*sr1* or *cpl1* (*esr3*) (data not shown). For cloning and heritability studies, an M3 *asr2* was out-crossed to the *Landsberg erecta* ecotype (*Ler*). All F_1_ plants showed the wild-type phenotype, and the F_2_ plants displayed a ~3:1 (wild-type:mutant) segregation suggesting the *asr2* phenotype was due to a recessive, monogenetic trait (59:21, χ^2^ test *p* = 0.80). 

### 2.2. Basal Defence Gene Expression is Altered in asr2

*GSTF8:LUC* expression was confirmed in the M3 *asr2* generation by in vivo quantification and a biochemical luciferase assay (Figure 1b). Quantitative real-time PCR (qRT-PCR) identified a small (2-fold) but non-significant increase in endogenous *asr2 GSTF8* expression relative to wild-type *GSTF8:LUC* seedlings (Figure 1c). Since enhanced pathogen or pest resistance in the *esr1* and *cpl1* mutants is linked to altered defence-associated pathway expression, we hypothesised that *asr2* may also be altered in its defence gene expression. We, therefore, assessed the expression of two defence marker genes; *Pathogenesis Related1* (*PR1*), an SA pathway marker; and *Plant Defensin1.2* (*PDF1.2*), a jasmonic acid (JA) pathway marker. In a simplistic model, defences dependent on the SA-signalling pathway are generally required for resistance against biotrophs, while the JA-signalling pathway is required for resistance against necrotrophic pathogens [18,19]. A small (2.3-fold) but non-significant increase in *PR1* expression was measured in *asr2* (Figure 1c). *PDF1.2* expression, on the other hand, was significantly decreased by nearly 12-fold compared to wild-type *GSTF8:LUC* plants (Figure 1c). This suggests a potential imbalance in *asr2* defence signalling.

**Figure 2:**
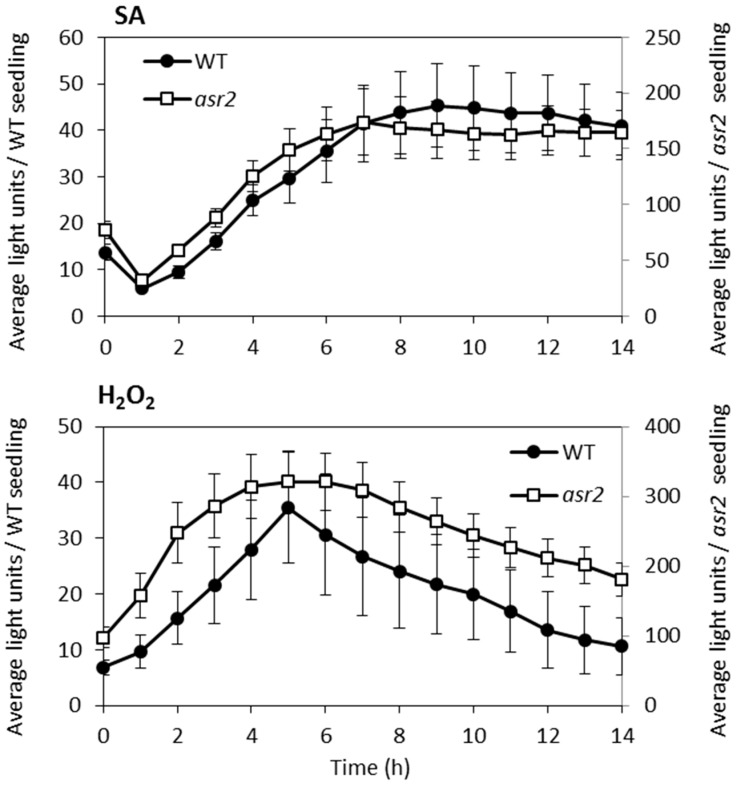
*GSTF8:LUC* response in *asr2* is maintained following salicylic acid (SA) or H_2_O_2_ induction. Average *GSTF8:LUC* expression per wild-type (WT) and *asr2* seedling per hour after treatment with 1 mM salicylic acid (SA) (upper panel) or 1 mM H_2_O_2_ (lower panel). Values are averages ± SE (*n* = 10) from four-day-old seedlings. WT values are plotted on the primary (left) axis and *asr2* values on the secondary (right) axis.

The higher levels of the SA-inducible *GSTF8* and *PR1* genes in *asr2* prompted us to determine whether *GSTF8:LUC* promoter activity in this mutant was hyper-responsive to SA [10,11,13]. Induction of *GSTF8:LUC* activity after SA treatment was seen in both wild-type and *asr2*, with the *asr2* mutant achieving a higher level of expression but a smaller fold change in activation (*asr2* 2.2-fold, and wild-type 3.3-fold). (Figure 2). A similar result was obtained following H_2_O_2_ treatment (wild-type 5.3-fold; *asr2* 3.3-fold). The lower fold change in *asr2* is likely due to its higher basal level of *GSTF8:LUC* expression and may represent an upper limit of activation potential of this promoter. In both treatments, the temporal *GSTF8:LUC* expression profile in *asr2* closely resembled that in wild-type. For subsequent assays, a twice-backcrossed (to wild-type) *asr2* line was developed to remove other ethyl methansulfonate (EMS)-induced mutations. This line had the same phenotypes as the M3 *asr2* line (data not shown).

### 2.3. ASR2 is Necessary for Resistance Against the Necrotrophic Fungal Pathogens Fusarium oxysporum and Alternaria brassicicola

**Figure 3:**
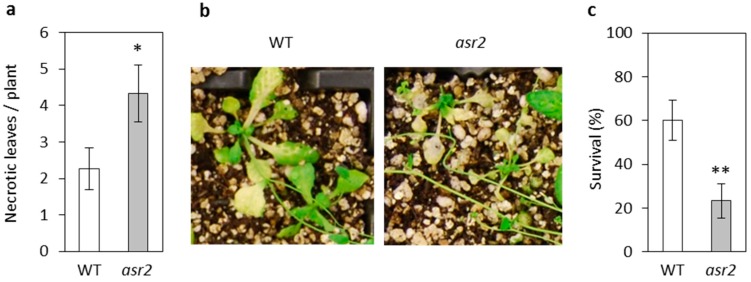
*asr2* has increased susceptibility to the necrotrophic fungal pathogen *Fusarium oxysporum*. (**a**–**c**) Disease phenotypes of *F*. *oxysporum* inoculated plants with (**a**) necrotic leaves and (**b**) representative images of plants at 14 days post inoculation (dpi), and (**c**) survival of plants at 21 dpi. Values are averages ± SE (*n* = 30). Asterisks indicate values that are significantly different (** *p* < 0.01, * *p* < 0.05 Student’s *t*-test) from wild-type (WT). Similar results were obtained in independent experiments.

**Figure 4:**
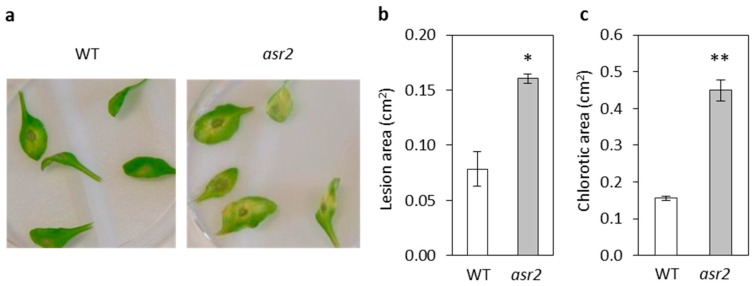
*asr2* had increased susceptibility to the necrotrophic fungal pathogen *Alternaria brassicicola*. (**a**–**c**) *A*. *brassicicola* induced lesions at 3 dpi with (**a**) representative images of leaves, (**b**) size of *Alternaria* induced lesions, and (**c**) size of chlorotic regions. Values are averages ± SE of three biological replicates consisting of lesions measured from five inoculated leaves per plant. Asterisks indicate values that are significantly different (** *p* < 0.01, * *p* < 0.05 Student’s *t*-test) from wild-type (WT). Similar results were obtained in independent experiments.

The *esr1* and *cpl1* mutants both had increased resistance to the root fungal pathogen *F. oxysporum*, and also showed altered responses to the leaf fungal pathogen *A. brassicicola*. We hypothesised that *asr2* may also display an altered response to these pathogens considering its down-regulated expression of the JA-marker gene *PDF1.2* and this gene’s association with resistance to necrotrophic fungal pathogens [20,21,22]. Following inoculation with *F. oxysporum*, disease symptom development progressed more quickly in *asr2* compared to wild-type plants. Within 14 days post inoculation (dpi), on average 5.3 leaves per *asr2* plant showed *Fusarium* disease symptoms compared to 3.8 leaves per wild-type plant. Of these diseased leaves, the number of necrotic leaves on *asr2* plants was significantly higher than the number on wild-type (Figure 3a,b). By 21 dpi, the survival rate of *asr2* plants was significantly lower than wild-type plants—23% compared to 60% (Figure 3c). On assessment of responses to *A. brassicicola*, significantly larger lesions developed on *asr2* leaves compared to wild-type (Figure 4a,b). The disease assay also induced chlorosis symptom development on inoculated leaves where the region of chlorosis on *asr2* plants was observed to also be significantly larger compared to wild-type (Figure 4b,c). Overall, the *Fusarium* and *Alternaria* disease phenotypes suggest *ASR2* is required for defence responses against necrotrophic fungal pathogens.

### 2.4. asr2 Has an Accelerated Flowering Phenotype

**Figure 5:**
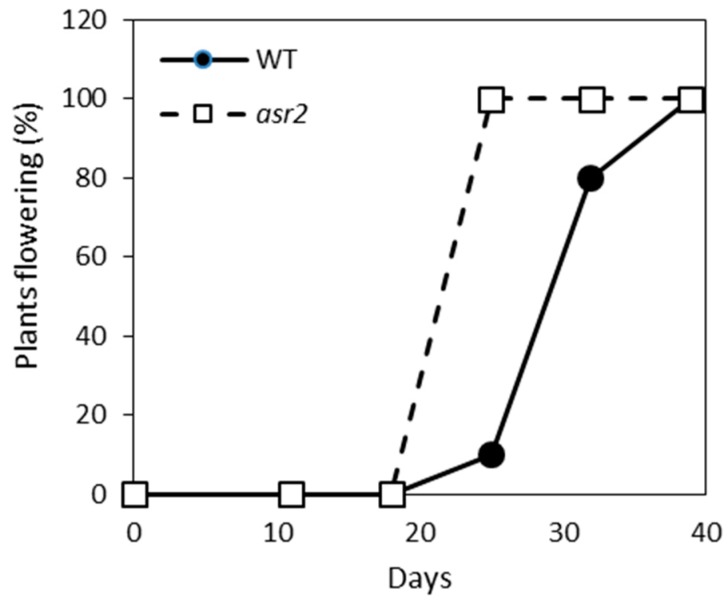
*asr2* has an early flowering phenotype. The number of flowering wild-type (WT) and *asr2* plants were measured over a 40-day time-course (*n* = 10). Similar results were obtained in an independent experiment.

Abnormalities in basal defence gene expression are often linked to altered developmental phenotypes or other pleiotropic effects (reviewed in References [23,24]). To determine if the imbalance in SA–JA defence gene expression was reflective of other abnormalities in *asr2* plants, we assessed their growth and flowering over an eight-week period. *asr2* plants were phenotypically normal apart from an early flowering phenotype. Within three and a half weeks post-germination, all *asr2* plants had transitioned to the flowering phase compared to only 10% of wild-type plants (Figure 5). A further two weeks were required for all wild-type plants to enter flowering.

### 2.5. Fine Mapping Localises asr2 to Four Genes on Chromosome 2

To map the loci encompassing *asr2* we employed a Next-Generation Mapping (NGM) approach that we had previously successfully used to map several *esr1* and *esr3/cpl1* alleles and narrow their causal loci to a handful of genes [8,16,25]. The process of NGM identifies the mutation of interest by quantifying the contribution of single nucleotide polymorphisms (SNPs) in a pooled F_2_ population between a mutant and its mapping population. We generated a mapping population between *asr2* (in Col-0 background) and the *Ler* ecotype and selected 60 homozygous *asr2* F_2_s based on their constitutive *GSTF8:LUC* phenotype. DNA was extracted from tissue pooled from the 60 F_2_s and sequenced on an Illumina HiSeq Platform. Reads were mapped to the Arabidopsis TAIR10 genome, and SNPs were identified and processed through the web-based NGM tool [25]. 

**Figure 6:**
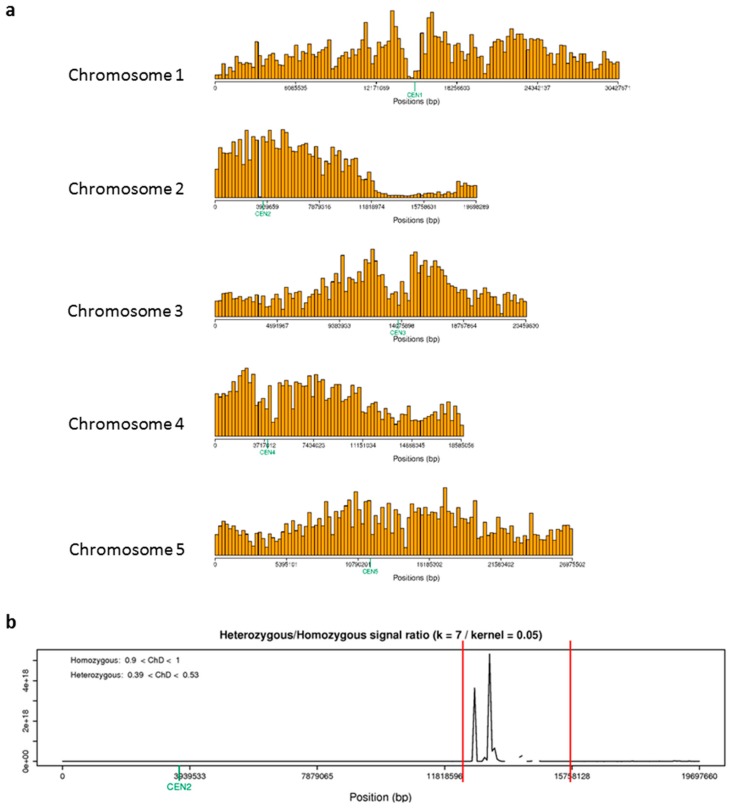
Fine mapping of *asr2* locates it to a region of chromosome 2. Next-Generation Mapping (NGM) was applied to an *asr2* mapping population by sequencing homozygous *asr2* F_2_ plants from an *asr2* and *Ler* outcross, and processing SNPs that deviated from the Arabidopsis TAIR10 genome reference sequence through the NGM tool http://bar.utoronto.ca/ngm/ [25]. (**a**) The tool identified SNP desserts (depression on chromosome 2) corresponding to *asr2* linkage. Shown are genome-wide SNP frequencies (y-axis) plotted as a function of chromosomal position (x-axis) using a bin size of 250 kb. (**b**) Closer inspection of chromosome 2 narrowed the *asr2* locus to a 2,093,087 bp region indicated by peaks on the y-axis (ratio of homozygous to heterozygous signals) and bordered by two red bars.

SNP desserts, corresponding to linkage to the *asr2* mutation, were identified on chromosome 2 (Figure 6a). The proportion of reads at a polymorphic genomic position that differed from the reference genome sequence was calculated via the NGM tool and a discordant chastity statistic provided. A discordant chastity statistic of 0 represents genomic positions where the reference base is observed, 0.5 for equal mutant and mapping parental genotype, and a value of 1.0 for all positions that are homozygous for a base that differs from the Col-0 genome, that is, an induced mutation. Closer inspection of chromosome 2 with a discordant chastity cut-off value of 0.85 narrowed the *asr2* locus to a 2,093,087 bp region that encompassed eight candidate mutations in coding regions (Figure 6b and Table 1). All mutations encoded non-synonymous codons with one encoding a stop codon change (At2g35170 Histone H3 K4-specific methyltransferase). These were At2g31320 *POLY(ADP-RIBOSE) POLYMERASE 1* (*PARP1*), At2g31400 *GENOMES UNCOUPLED 1* (*GUN1*), At2g31990 an Exostosin family protein, At2g33170 a Leucine-rich repeat receptor-like protein, At2g35170 a Histone H3 K4-specific methyltransferase, At2g35740 *NOSITOL TRANSPORTER 3* (*ATINT3/INT3*), At2g36420 *TON1 RECRUITING MOTIF 27* (*TRM27*), and At2g36835 an unknown protein.

We undertook the same genetic complementation studies used to clone the *esr1* and *esr3/cpl1 alleles* [8,16] by searching for publicly available mutant or T-DNA knockout lines in the candidate *ASR2* genes. Firstly, a search of the SALK Arabidopsis T-DNA insertion collection identified insertions in coding regions of three candidate genes. These were At2g33170 Leucine-rich repeat receptor-like, At2g36420 TRM27, and At2g36835 encoding the unknown protein. No SALK insertion lines in the coding regions for the five remaining candidates were available. We obtained homozygous lines of the three available coding region mutants and challenged them with *F. oxysporum* which provided a strong and relevant phenotype representative of the *asr2* mutant. The SALK_092719C insertion line in Leucine-rich repeat receptor-like had poor germination, and we were unable to successfully screen for disease response. Neither of the two other T-DNA lines (SALK_049785C, At2g36420, TRM27; SALK_043358C, At2g36835, unknown protein) differed from wild-type plants in their *F. oxysporum* disease phenotype (diseased plants *P* > 0.05), suggesting *ASR2* is not encoded by these candidate genes.

Of the five candidates for which no SALK T-DNA knockout lines were available, we ranked At2g31400 *GUN1* as a strong candidate as it encodes a member of the RNA-binding pentatricopeptide-repeat protein family which are involved in RNA processing roles related to the functions of ESR1 and ESR3/CPL1. For example, pentatricopeptide-repeat proteins can facilitate processing, splicing, editing, stability, and translation of RNAs [26]. We searched the literature and obtained an EMS-induced stop-codon *gun1* mutant termed *gun1-9* [27]. Screening of *gun1-9* with *F. oxysporum* did not reveal enhanced susceptibility that mimicked the *asr2* phenotype (diseased plants *P* > 0.05). We also undertook genetic complementation studies with *gun1-9*. The two *asr2* and *gun1-9* homozygous recessive mutants were crossed, and the F_1_ progeny screened for complementation of the constitutive *GSTF8:LUC* phenotype. Neither of the reciprocal crosses between *asr2* and *gun1-9* displayed constitutive luciferase expression (data not shown), suggesting a wild-type copy of *ASR2* in *gun1-9* had restored the wild-type *GSTF8:LUC* phenotype and that *ASR2* equivalently does not encode *GUN1*. For the four remaining candidate genes for which no suitable mutant lines were available, molecular complementation tests have been prioritised.

## 3. Discussion

Facilitated through a forward genetic screen for mutants altered in *GSTF8:LUC* defence/stress reporter expression, we previously identified several alleles of the *ESR1/KH-domain* and *CPL1* genes that conferred constitutive *GSTF8:LUC* activity and enhanced resistance to fungal pathogens. In this study, we identified *asr2* as a second class of mutant that also conferred constitutive *GSTF8:LUC* activity but was more susceptible than wild-type to necrotrophic fungal pathogens—the root-infecting pathogen *F. oxysporum* and the leaf-infecting pathogen *A. brassicicola*.

The *asr2* plants displayed contrasting expression of two classical SA- or JA-mediated defence marker genes where the JA-marker gene *PDF1.2* was highly down-regulated. A functional JA-pathway is required for resistance against *A. brassicicola* [22,28], and the *asr2 PDF1.2* phenotype may contribute to its susceptibility towards this pathogen. PDF1.2 is also associated with resistance against *F. oxysporum*. For example, in ERF4 transcription factor overexpression lines, their impairment in JA-induced *PDF1.2* expression is linked to increased *F. oxysporum* susceptibility [29]. JA signalling can however also play contrasting roles in *F. oxysporum* disease outcome. It is proposed that non-defensive aspects of JA-signalling such as senescence processes strongly contribute [30,31,32,33,34]. Consequently, mutants with reduced defensive and non-defensive JA-signalling such as *esr1-1* are more resistant [8]. This disease-resistance phenotype is even more pronounced in mutants with abolished or severely impaired JA-signalling such as the *coronatine insensitive1 (coi1)* or *mediator* complex mutants *med25/pft1*, *med18,* or *med20* [30,32,33].

The *asr2* mutant also displayed an early flowering phenotype. A significant correlation between flowering time and *F. oxysporum* disease outcome was established through disease assays on a large Arabidopsis ecotype collection that differed in their flowering response [35]. Early flowering ecotypes were more susceptible while late-flowering ecotypes showed enhanced resistance. Increased expression of the negative regulator of flowering time *FLOWERING LOCUS C* (*FLC*) in *cpl1* and several *med* mutants was linked to their delayed flowering and enhanced disease resistance [16,30,33]. However, a positive correlation between reduced *FLC* expression (accelerated flowering time) and increased susceptibility to *F. oxysporum* did not hold true [35]. Once the *ASR2* gene is cloned, functional studies using *ASR2* knockout or *ASR2* overexpression lines will be used to dissect the role of ASR2 in defence and flowering.

Next-Generation Mapping narrowed the causal *asr2* loci to eight candidate genes. *Fusarium* disease assays on publicly available EMS or SALK T-DNA insertion mutants eliminated at least three genes as candidates. Of the remaining candidates, POLY(ADP-RIBOSE) POLYMERASE 1 (PARP1) is one of three PARPs in Arabidopsis whose activity is activated in response to stress such as ROS. Their post-translational modification of nuclear proteins activates DNA repair machinery (reviewed in Reference [36]). The defined role of PAR1 in stress is unclear as plants with reduced PAR activity by means of chemical inhibitors or gene silencing are tolerant of a broad range of abiotic stresses, but individual, double, or triple *PARP* GABI-Kat knockout lines do not differ from wild-type [36,37]. A *parp1/parp2* double mutant is slightly more susceptible to virulent *Pseudomonas* bacteria [38]. The individual role of PARP1 and PARP2 in this response was not tested due to potential functional redundancy. However, the minor *Pseudomonas* susceptibility response in the double mutant suggests individual mutants may show no phenotype. *NOSITOL TRANSPORTER 3* (*ATINT3/INT3*) may affect stress responses. The *Brassica napus* homologue of this gene is up-regulated following infection with the necrotrophic fungal pathogen *Sclerotinia sclerotiorum* [39]. The two remaining *ASR2* candidate genes, exostosin family protein and histone H3 K4-specific methyltransferase, are functionally uncharacterised and to our knowledge, no link to biotic stress has been reported. Of the detected SNPs in the mapped *asr2* loci, the mutation in At2g35170 (histone H3 K4-specific methyltransferase) is the only predicted stop codon change. Based on the above characteristics, PARP1, ATINT3, and the methyltransferase have been prioritised for molecular complementation studies in the *asr2* background, and the *parp1/parp2* T-DNA double mutant will be obtained for *Fusarium* disease assessment.

In summary, the high-throughput *GSTF8:LUC* forward genetic screen facilitated the discovery of the *asr2* mutant accelerated in flowering and heightened in susceptibility to root- and leaf-infecting necrotrophic fungal pathogens. The susceptibility to pathogens of dissimilar lifecycles suggests ASR2 is an important component of the host defence response and its functional characterisation will be important to our combined knowledge of plant disease resistance mechanisms.

## 4. Materials and Methods 

### 4.1. Plant Material and Growth Conditions

The *Arabidopsis thaliana* Columbia-0 transgenic line (JC66/*GSTF8:LUC*) containing 791 bp of the *GSTF8* promoter fused to a luciferase reporter [12,15] was used in all experiments unless otherwise noted. Agar plate assays were performed using surface-sterilised seeds plated onto Murashige and Skoog (MS) salt agar plates as described previously and supplemented with 50 uM luciferin (Biosynth AG) for luciferase assays [6,11]. Plate- and soil-grown plants were incubated under a 16-h light/8-h dark cycle at 22 °C.

### 4.2. Bioluminescence and Luciferase Assays

Biochemical luciferase assays and imaging of in vivo seedling bioluminescence in an EG & G Berthold molecular light imager were conducted as described previously [6,8,15]. 

### 4.3. Genetic and Mapping Studies

For allelism tests, reciprocal crosses between *asr2* and *esr1* or *esr3/cpl1* plants were conducted. Bioluminescence activity in progeny was assessed. Next-Generation mapping was performed as described previously [8]. Briefly, a two-times backcrossed *asr2* line was crossed with *Ler*, and pooled DNA (CTAB extraction) from 60 homozygous *asr2* F_2_ plants (exhibiting constitutive *GSTF8:LUC* activity) were sequenced by the Australian Genome Research Facility (AGRF) using an Illumina HiSeq Platform to provide 9.25 Gb of raw data that would be sufficient to provide ~50× genome coverage. A total of 85.9 million paired-end reads (100 bp in length) were cleaned, trimmed, and mapped to the Arabidopsis TAIR10 genome reference sequence, SNPs called using the recommended SAMtools mpileup script, and processed through the NGM tool http://bar.utoronto.ca/ngm/ [25].

### 4.4. Pathogen and Flowering Time Assays

Fungal pathogen inoculations were performed according to Thatcher et al. and Gleason et al. [7,32]. For *F. oxysporum* (Fo5176), root-dip inoculations on 4-week-old plants were carried out using a 1×10^6^ cell/mL spore suspension. For *A. brassicicola* (UQ4273), a 1×10^6^ cell/mL spore suspension was applied to leaves of 3- to 4-week-old plants. Lesion size was measured with ImageJ [40]. Flowering time assays were conducted under long day conditions of a 16-h light/8-h dark cycle at 22 °C (*n* = 10).

### 4.5. RNA Isolation and qRT-PCR

For qRT-PCR experiments, tissue was collected from whole 7-day-old seedlings germinated and grown on MS media. Two separate biological replicates were taken for each genotype, with each replicate consisting of tissue pooled from 20 seedlings grown at the same time in the same environment, then frozen in liquid nitrogen and stored at −80 °C. RNA extractions, DNase treatment, cDNA synthesis, and qRT-PCR was performed on a MyiQ (Bio-Rad) system as described previously [6,14]. Gene expression was calculated using the *Elongation Factor1* reference gene (*EF-1-F* 5’ ATGCCCCAGGACATCGTGATTTCAT 3’; *EF-1-R* 5’ TTGGCGGCACCCTTAGCTGGATCA 3’) according to the equation: relative ratio gene of interest/EF-1 = (2^−Ct gene^)/(2^−Ct EF-1^) where Ct is the cycle threshold value. Gene-specific primer sequences used are *GSTF8* (*GSTF8-F* 5’ CCCCGTCGATATGAGAGC 3’; *GSTF8-R* 5’ GAGAGAGGGTCACTACTGCTTCTGG 3’), *PR1* (*PR1-F* 5’ TTCTTCCCTCGAAAGCTCAA 3’; *PR1-R* 5’ AAGGCCCACCAGAGTGTATG 3’) and *PDF1.2* (*PDF1.2-F* 5’ TGTTCTCTTTGCTGCTTTCGACG 3’; *PDF1.2-R* 5’ GCATGATCCATGTTTGGCTCCT 3’).

## Figures and Tables

**Table 1:** Candidate *asr2* genes. Shown are predicted EMS-induced mutations in coding regions of genes in the *asr2* loci. Reference base refers to the Col-0 reference and SNP base to the detected SNP. DC refers to the discordant chastity value.

Chrom.	Position	Ref. Base	SNP Base	Depth	DC	Accession	Strand	Ref. Codon	SNP Codon	AA Change
2	13358132	C	T	55	0.91	AT2G31320	-	GGA	GAA	G- > E
2	13389698	C	T	57	0.98	AT2G31400	-	GAG	AAG	E- > K
2	13612057	C	T	41	0.92	AT2G31990	-	GCT	ACT	A- > T
2	14057731	C	T	46	0.91	AT2G33170	-	GGA	GAA	G- > E
2	14829201	C	T	20	0.85	AT2G35170	+	CAA	TAA	Q- > *
2	15025500	C	T	32	0.93	AT2G35740	-	GGT	AGT	G- > S
2	15288977	C	T	49	0.96	AT2G36420	+	TCT	TTT	S- > F
2	15451219	G	A	41	0.95	AT2G36835	-	CCG	CTG	P- > L
